# Combination Therapy Using Low-Concentration Oxacillin with Palmitic Acid and Span85 to Control Clinical Methicillin-Resistant *Staphylococcus aureus*

**DOI:** 10.3390/antibiotics9100682

**Published:** 2020-10-08

**Authors:** Hun-Suk Song, Tae-Rim Choi, Shashi Kant Bhatia, Sun Mi Lee, Sol Lee Park, Hye Soo Lee, Yun-Gon Kim, Jae-Seok Kim, Wooseong Kim, Yung-Hun Yang

**Affiliations:** 1 Department of Biological Engineering, College of Engineering, Konkuk University, Hwayang-dong, Gwangjin-gu, Seoul 05029, Korea; shs9736@konkuk.ac.kr (H.-S.S.); srim1004@konkuk.ac.kr (T.-R.C.); shashikonkukuni@konkuk.ac.kr (S.K.B.); dltjsal6845@konkuk.ac.kr (S.M.L.); shckd2020@konkuk.ac.kr (S.L.P.); lhs2265696@konkuk.ac.kr (H.S.L.); 2 Institute for Ubiquitous Information Technology and Applications (CBRU), Konkuk University, Seoul 05029, Korea; 3 Department of Chemical Engineering, Soongsil University, 511 Sangdo-dong, Seoul 156-743, Korea; ygkim@ssu.ac.kr; 4 Department of Laboratory Medicine, Kangdong Sacred Heart Hospital, Hallym University College of Medicine, Seoul 05355, Korea; jaeseok@hallym.ac.kr; 5 College of Pharmacy and Graduate School of Pharmaceutical Sciences, Ewha Womans University, Seoul 03760, Korea; wooseong_kim@ewha.ac.kr

**Keywords:** MRSA, drug combination therapy, palmitic acid, span85

## Abstract

The overuse of antibiotics has led to the emergence of multidrug-resistant bacteria, such as methicillin-resistant *Staphylococcus aureus* (MRSA). MRSA is difficult to kill with a single antibiotic because it has evolved to be resistant to various antibiotics by increasing the PBP2a (*mecA*) expression level, building up biofilm, introducing SCCmec for multidrug resistance, and changing its membrane properties. Therefore, to overcome antibiotic resistance and decrease possible genetic mutations that can lead to the acquisition of higher antibiotic resistance, drug combination therapy was applied based on previous results indicating that MRSA shows increased susceptibility to free fatty acids and surfactants. The optimal ratio of three components and the synergistic effects of possible combinations were investigated. The combinations were directly applied to clinically isolated strains, and the combination containing 15 μg/mL of oxacillin was able to control SCCmec type III and IV isolates having an oxacillin minimum inhibitory concentration (MIC) up to 1024 μg/mL; moreover, the combination with a slightly increased oxacillin concentration was able to kill SCCmec type II. Phospholipid analysis revealed that clinical strains with higher resistance contained a high portion of 12-methyltetradecanoic acid (anteiso-C15:0) and 14-methylhexadecanoic acid (anteiso-C17:0), although individual strains showed different patterns. In summary, we showed that combinatorial therapy with a low concentration of oxacillin controlled different laboratory and highly diversified clinical MRSA strains.

## 1. Introduction

Over decades, the overuse of antibiotics has brought about multidrug-resistant bacteria [1]. Among these, methicillin-resistant *Staphylococcus aureus* (MRSA) is difficult to treat in communities and healthcare facilities owing to its quick spread and multidrug resistance [2]. To be more specific, they acquired β-lactam antibiotic resistance through the repressing Agr quorum-sensing circuit followed by *mecA* expression which competes with virulence factors in MRSA [3]. Thus, a higher level of antibiotic resistance or virulence regulation is obtained by the Agr quorum-sensing circuit with expression control [4].

Oxacillin is a penicillinase-resistant β-lactam antibiotic that has replaced methicillin in clinical use [5]. Because oxacillin is resistant to penicillinase enzymes, such as those produced by *S. aureus*, it is widely used clinically to treat penicillin-resistant *S. aureus*. With the widespread use of oxacillin, oxacillin-resistant MRSA has become prevalent. Specifically, MRSA has acquired drug resistance by the horizontal transfer of resistance genes in the staphylococcal cassette chromosome *mec* [6]. In other words, it is represented as SCCmec type based on its genetic variants conferring antibiotic resistance. Among these, SCCmec types II and III are classified as healthcare-associated MRSA which are resistant to various kinds of antibiotics due to their genetic and physical traits. Therefore, the use of a single antibiotic for the treatment of *S. aureus* is not often recommended owing to their multi-resistance mechanism [7]. As a possible solution, drug combination is considered a simple method to avoid the complex resistance mechanism of *S. aureus* [8]. This is because multiple antibacterial agents, such as antibiotics, free fatty acids, flavonoids, and surfactants, can exert synergistic effects arising from the complex activities of their combinations [9,10].

Agents other than antibiotics, such as free fatty acids, polyunsaturated fatty acids (PUFAs), flavonoids, triclosan, and surfactants, have been found to evade the bacterial resistance mechanism [9,11,12,13]. Among these, free fatty acids are natural antibacterial compounds found in human tissues and fluids [14]. They exert antibacterial activities by disrupting the electron transport chain, interfering with oxidative phosphorylation, inducing the leakage of cell metabolites, inhibiting fatty acid biosynthesis, blocking enzymatic activity, and forming peroxidation products [15]. Palmitic acid (16:0, PA) is the most common saturated fatty acid found in the human body and it can be derived from foods or synthesized endogenously from other building blocks [16]. Depending on the condition, palmitic acid has shown effectiveness against MRSA. Specifically, palmitic acid at a concentration of over 1.6 mg/mL inhibited MRSA in vitro, and it inhibited the growth of MRSA in infected cells in vivo [15]. Moreover, in a previous study, palmitic acid was shown to eliminate the biofilm of MRSA and increase the effectiveness of oxacillin [9].

Surfactants, including polysorbate 80 and sodium alkyl sulfate, are compounds used for the disinfection of MRSA; they can inhibit biofilm formation and increase the effect of benzalkonium chloride, respectively [17,18]. In addition, surfactants can increase cell membrane permeability and decrease cell surface hydrophobicity. Sorbitan trioleate (Span85) is a non-ionic detergent used as an emulsifier in cosmetics, medicines, and nanoparticles and contains three oleic acid chains per molecule compared to palmitic acid [19]. Span85 has also shown effectiveness against MRSA by inhibiting biofilm formation [9].

Thus, we examined the possibility of combination therapy, selecting factors based on our previous study, including oxacillin, palmitic acid, and span85, and found that the optimization of factors in combination therapy could decrease the concentration of antibiotics, which would decrease the emergence of multidrug-resistant bacteria. Response surface methodology was introduced in this study to develop a combination therapeutic strategy using Δ*agr* mutant as a model organism, as the resistance of healthcare-associated MRSA (HA-MRSA) is acquired through the repression of the Agr operon. In other words, clinical isolates of MRSA generally fall into HA-MRSA, so the strain without the *agr* gene was chosen for the model study [20]. Subsequently, clinically isolated MRSA strains were treated with drug combinations under optimal conditions.

## 2. Results and Discussion

### 2.1. Inhibitory Effect of Palmitic Acid and Span85

**Figure 1:**
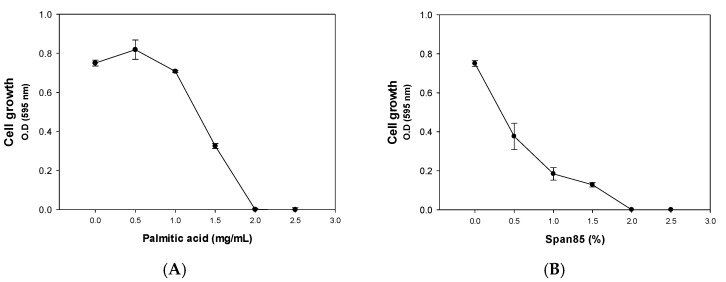
Effect of different levels of (**A**): palmitic acid and (**B**): span85 on the Δ*agr* strain. Error bars represent the standard deviation of three replicates.

In a previous study, we found that palmitic acid, which is the most common saturated fatty acid found in animals, plants, and microorganisms, as well as a component in soap, was capable of inhibiting biofilm formation of community-associated MRSA (CA-MRSA) LAC and its Δ*agr* mutant, which has a higher oxacillin minimum inhibitory concentration (MIC) (>200 μg/mL) [9]. Concomitant treatment with palmitic acid and oxacillin led to a dramatic increase in the efficacy of oxacillin. Similarly, span85, which is mainly used in medicines, cosmetics, textiles, paints, and petroleum as an emulsifier, thickener, anti-rust agent, and biodegradable surfactant based on a natural fatty acid (oleic acid) and sugar alcohol (sorbitol), eliminated the biofilm of the MRSA strains and decreased the MIC of oxacillin on MRSA. However, the effect of palmitic acid and span85 was investigated only at fixed concentrations, and the combinatorial effect was not investigated for possible applications. Considering that soaps contain more than 10% palmitic acid and that span85 is used at a concentration of 0.5–5% in drugs and cosmetics, these compounds could be used to control resistant bacteria causing several skin issues. In an initial analysis, the effects of different concentrations of palmitic acid and span85 were tested for dose-dependency; furthermore, Δ*agr* mutant, which is more resistant than the LAC MRSA strain, was used to evaluate the antibacterial activity of palmitic acid and span85. The results showed that palmitic acid at concentrations lower than 1 mg/mL exerted no significant effect on the growth of the Δ*agr* mutant cells (Figure 1A). However, it started to inhibit the cell growth when the concentration was over 1 mg/mL. Span85 also inhibited the growth of the Δ*agr* mutant cells at concentrations over 0.1% (*v*/*v*) (Figure 1B). Collectively, the MICs for each antibacterial compound are calculated (oxacillin = 256 μg/mL, palmitic acid = 2 mg/mL and span85 = 2% (*v*/*v*) ≈ 19.14 mg/mL)

### 2.2. Response Surface Methodology Analysis to Study the Effect of the Interaction of Different Antibacterial Agents

The advantage of combination therapies is the reduction in the antibiotic concentration used, as multiple activities can better attenuate or evade the antibiotic-resistance mechanisms of pathogenic bacteria. Response surface methodology analysis using the Box–Behnken design was introduced to set up the optimal concentration of three antibacterial agents to effectively eliminate MRSA [21,22,23]. Using concentrations higher than the MIC of each compound is meaningless; thus, the MIC_50_ of each agent was selected for the Box–Behnken design using Minitab 18 software to analyze the interaction and examine the desired response. The three significant variables, namely oxacillin, palmitic acid, and span85, were investigated with the values shown in Table 1 based on the diagonal sampling method [24,25].

To monitor the effect of the oxacillin concentration, we selected three values with different concentrations of oxacillin. The range of the oxacillin concentration changed from 0 to 100 μg/mL. The experimental design and results are shown in Table 2. The regression equation obtained after analysis of variance gave the response (optical density, 595 nm) as a function of three significant variables. To obtain a polynomial equation, a quadratic model was conducted to fit the data by least squares, and all terms, regardless of their significance, were included in the following equation (1):

Optical density (595 nm) = 0.792292 − 0.00576583X_1_ − 1.14626X_2_ + 0.575X_3_ + 0.36603X_2_^2^ + 0.00565111X_1_X_2_ − 0.00884X_1_X_3_ (1)

where X_1_: oxacillin, X_2_: palmitic acid, and X_3_: span85.

**Figure 2:**
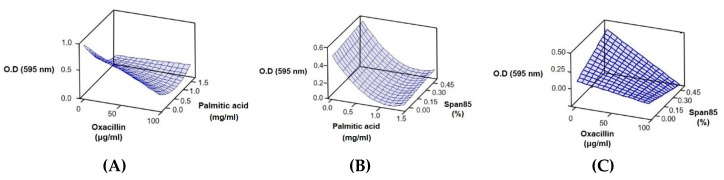
Plots showing the synergistic effect of oxacillin/palmitic acid/span85 on cell growth. (**A**) Oxacillin (μg/mL), palmitic acid (mg/mL); (**B**) palmitic acid (mg/mL), span85 (%); (**C**) oxacillin (μg/mL), span85 (%).

A surface plot for oxacillin, palmitic acid, and span85 is shown in Figure 2. Analysis of variance of the selected response showed a *p* value of <0.05, which indicated that the designed model was appropriate (Table 3). Surface plots showed that the concentrations of oxacillin and palmitic acid were important for the bactericidal effect (Figure 2A–C). Compared with the single use of span85 (Figure 1B), the combined use of span85 with a low concentration of palmitic acid or oxacillin incurred an antagonistic effect (Figure 2B,C).

### 2.3. Effect of Combined Therapy on the Δagr Strain and Clinically Isolated Strains

Once the concentrations of the three significant variables (oxacillin, palmitic acid, and span85) were set with the Δ*agr* mutant by response surface methodology analysis and response optimizer, we treated clinically isolated strains with the different combination therapies (Table 4). Low to high concentrations of oxacillin were considered. The focus was on the low concentrations, as antibiotic overuse has led to the emergence of multidrug resistance in MRSA. Thus, we examined whether the selected conditions were effective even for clinical isolates.

**Figure 3:**
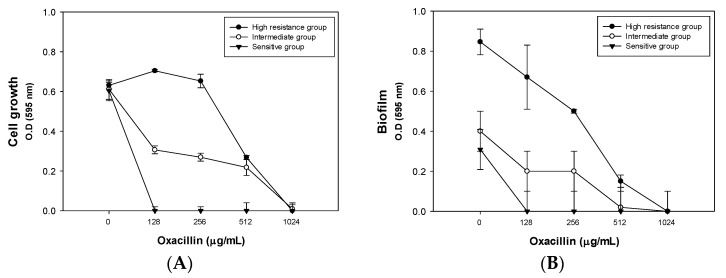
Effect of oxacillin on clinically isolated strains. (**A**) Effect of different oxacillin concentrations on the cell growth of clinical strains. (**B**) Effect of different oxacillin concentrations on the biofilm formation of clinical strains. The strains were classified into three groups: the high-resistance (MRSA8471 and MRSA9291), intermediate-resistance (MRSA2065, MRSA6288, MRSA7557, MRSA12779, MRSA14278, and MRSA14459), and sensitive (MRSA6230 and MRSA7875) groups. The error bars represent the standard deviation of three replicates for all strains. The averages of cell growth and biofilm for each group were calculated, and the results are simplified in the figures.

Except for MRSA6230 and MRSA14459, the other MRSA strains were HA-MRSA, having SCCmec type II and type III, but all clinical strains were found to have an oxacillin MIC of over 128 μg/mL (Figure 3A). The MIC for each strain is listed in Table 5. Multilocus sequence typing and spa typing, which were determined in the previous study, are also included in the table [26,27]. In addition, biofilm formation was compared between the different oxacillin concentrations (Figure 3B). Depending on the degree of their antibiotic resistance, the clinical isolates were classified into high-resistance (MRSA8471 and MRSA9291), intermediate-resistance (MRSA2065, MRSA6288, MRSA7557, MRSA12779, MRSA14278, and MRSA14459), and sensitive (MRSA6230 and MRSA7875) groups.

**Figure 4:**
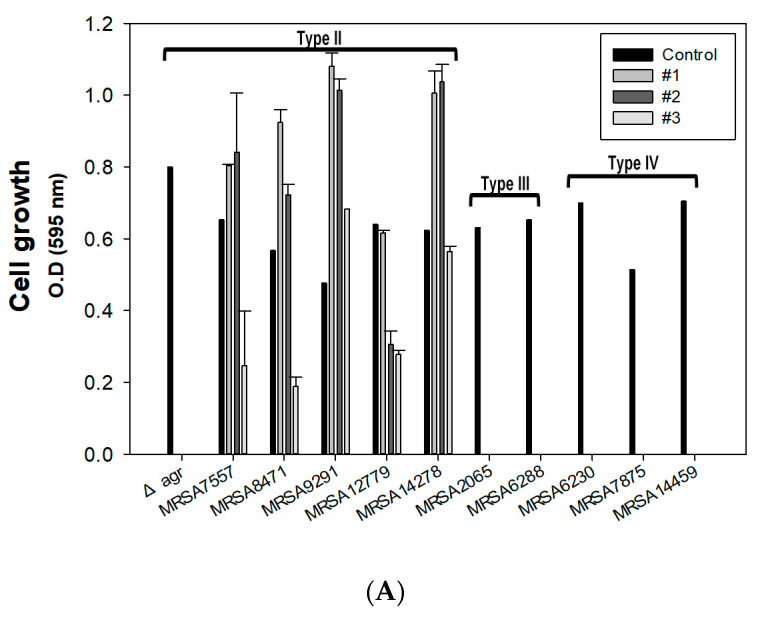

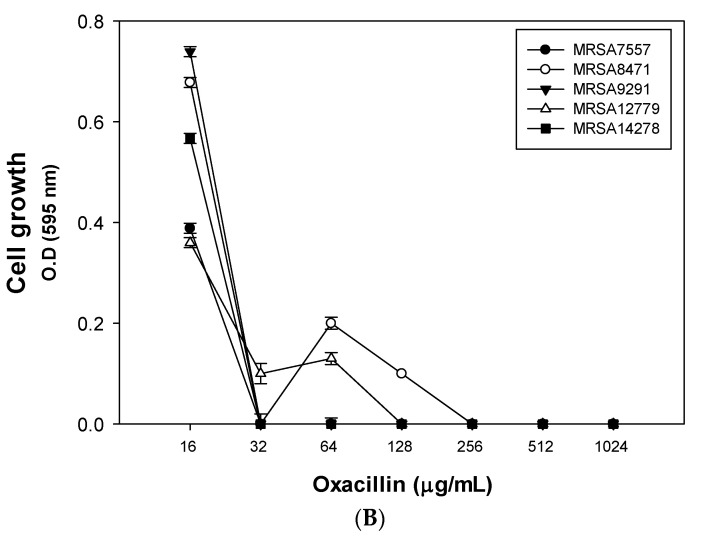
Validation of optimized condition and MIC calculation for clinical isolates with a high level of antibiotic resistance. (**A**) The effect of combination therapy on methicillin-resistant *Staphylococcus aureus* (MRSA) using optimized condition #1 (oxacillin = 15 μg/mL, palmitic acid = 1.3 mg/mL, and span85 = 0.1% (*v*/*v*)), #2 (oxacillin = 50 μg/mL, palmitic acid = 1 mg/mL, and span85 = 0.08% (*v*/*v*)), and #3 (oxacillin = 100 μg/mL, palmitic acid = 0.3 mg/mL, and span85 = 0.4% (*v*/*v*)). (**B**) Treatment of the clinically isolated SCCmec type II strains using condition #1 (palmitic acid = 1.3 mg/mL and span85 = 0.1% (*v*/*v*)) with variations in the oxacillin concentration. The error bars represent the standard deviation of three replicates.

As a control experiment, we found that all three conditions were sufficient to kill the Δ*agr* mutant, which mimics the characteristics of HA-MRSA with attenuated virulence. Additionally, the growth of MRSA2065, MRSA6230, MRSA6288, MRSA7875, and MRSA14459 was totally inhibited by all three combinations (Figure 4A). All clinical SCCmec type III and IV strains have a much higher MIC of oxacillin of more than 128 μg/mL. This makes it difficult to kill MRSA with a low concentration of oxacillin. However, our combination therapy sets were able to kill five different strains, even with 15 μg/mL of oxacillin. Though the combination therapy was set up with the Δ*agr* mutant strain, the results showed that it was still enough to kill the clinical SCCmec type III and IV strains. However, the oxacillin concentration used was not enough to eliminate MRSA7557, MRSA8471, MRSA9291, MRSA12779, and MRSA14278, which are found to be SCCmec type II strains, which exhibit multidrug resistance with an oxacillin MIC level of about 1024 μg/mL. Killing assays were carried out once again after the confirmation of the oxacillin MIC; the results confirmed that even the SCCmec type II clinical isolates could be killed with 256 μg/mL of oxacillin (Figure 4B). The clinical isolates contained a high ratio of odd anteiso-fatty acids in the membrane (data not shown), and the biofilm was thicker in the clinical isolates [28]. The individual treatment of the clinical strains with palmitic acid and span85 showed a similar pattern to the Δ*agr* mutant (Figure 1), but with different values. However, the combination of the three components decreased the amount of each component.

### 2.4. Characterization of Clinically Isolated strains with Phospholipid Fatty Acid (PLFA) Analysis

**Figure 5:**
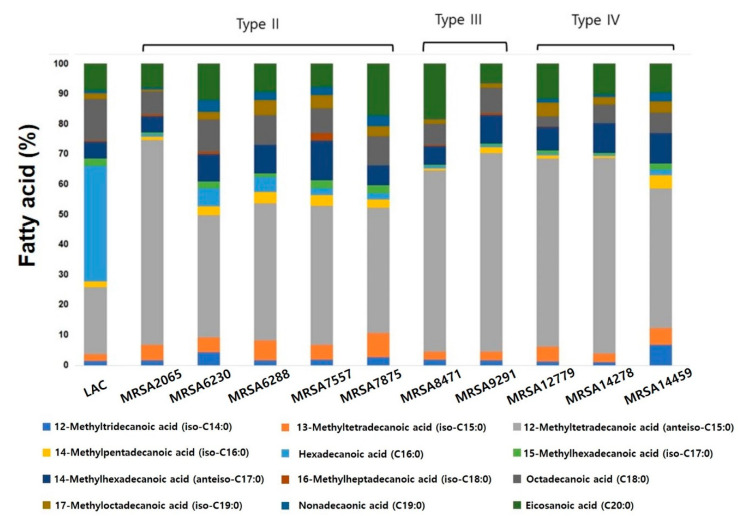
Fatty acid analysis of LAC and clinically isolated strains.

MRSA strains with higher antibiotic resistance tend to have extraordinary features, such as compositional changes in membrane lipid, biofilm formation, persistent cell formation, stable membrane integrity for membrane microdomain assembly for optimal oligomerization of PBP2a, high *mecA* expression, and increased cell surface hydrophobicity [29,30]. To elucidate the reason for the different effects of our combinations, we performed phospholipid fatty acid (PLFA) analysis because fatty acid composition in the cytoplasmic membrane can affect the antibiotic resistance of pathogenic bacteria [9,31]. PLFA analysis showed that a major portion of phospholipids in the clinical MRSA strains contained abundant 12-methyltetradecanoic acid (anteiso-C15:0) and 14-methylhexadecanoic acid (anteiso-C17:0) instead of hexadecanoic acid (C16:0) (Figure 5). It is known that methyl branching modifies the thermotropic behavior and enhances the fluidity of lipid bilayers. It reduces lipid condensation, decreases the bilayer thickness, and lowers chain ordering and formation of kinks at the branching point [32]. Highly resistant SCCmec type II strains appeared to show different PLFA compositions, except for MRSA7557, showing a lower amount of 12-methyltetradecanoic acid (anteiso-C15:0) than type III and IV strains and a relatively higher amount of 13-methyltetradecanoic acid (iso-C15:0) and hexadecanoic acid (C16:0), although it was difficult to link this to the increased resistance of SCCmec type II strains. Although SCCmec type II strains showed different results than those of type III and IV, these results in clinical strains showed the potential of combination therapy by decreasing oxacillin concentration with the same antibiotic activity.

## 3. Materials and Methods

### 3.1. Bacterial Strains, Media, and Culture Conditions

For cell preparation, the Δ*agr* mutant [33] was cultured in tryptic soybean broth (TSB) agar and/or liquid broth. For pre-culture, a single colony of the strain from a TSB agar plate was used to inoculate 5 mL of TSB medium. Next, 1% (*v*/*v*) of the cell culture suspension was inoculated in a 96-well plate for the antibiotic resistance test, and the cells were cultivated overnight in an incubator at 37 °C without shaking unless stated otherwise.

### 3.2. Antibacterial Agents

Oxacillin, palmitic acid, and span85 were purchased from Sigma-Aldrich (St. Louis, MO, USA). Stock solutions of these agents were prepared at various concentrations in sterile dimethyl sulfoxide.

### 3.3. Analysis of Cell Growth and Biofilm Formation

Cell growth was measured at 595 nm using a 96-well microplate reader (TECAN, Männedorf, Switzerland). Biofilm formation was analyzed by crystal violet staining according to a previously described protocol [9]. Briefly, the supernatant was aspirated. The biofilm was then fixed with methanol, air-dried, and stained with 200 μL of 0.2% crystal violet solution for 5 min. Next, the crystal violet solution was removed, and the biofilm was washed with distilled water and air-dried. Finally, the optical density of the biofilm was detected at 595 nm using a 96-well microplate reader.

### 3.4. Response Surface Methodology Analysis

After selecting the optimal concentrations for oxacillin, palmitic acid, and span85, combination therapies were optimized using Minitab software 18 (Minitab, State College, PA and SPSS, IBM Corp. 2011, Version 18, Armonk, NY, USA) through the Box–Behnken design and response surface methodology analysis. Experiments were conducted in triplicate, and the cell growth of MRSA was determined. Coefficients were determined using the experimental values using the full quadratic model f(x, y, z) = (x, y, z) = ax^2^ + by^2^ + cz^2^ + dxy + eyz + fxz + gx + hy + iz + j, (a,b,c ≠ 0). Using surface plots, the relationships between the variables were investigated and validated.

### 3.5. PLFA Analysis

Briefly, 10 mL of the liquid culture was cultivated in TSB with 1% inoculum in an incubator at 37 °C with shaking at 200 rpm. Samples were collected at 8 and 16 h. Next, the samples were centrifuged at 3500 rpm for 20 min, and total fatty acids were extracted with 0.15 M citric acid buffer/chloroform/methanol (7:7.5:5, *v*/*v*/*v*) and incubated in an incubator at 37 °C with shaking at 200 rpm for 2 h. The chloroform phase was collected, and the chloroform was slowly evaporated under compressed N_2_ to avoid oxidation. The sample was loaded into a sialic acid column and then serially eluted with 5 mL each of chloroform, acetone, and methanol. The methanol phase was collected for PLFA analysis. Next, 1 mL of toluene was added to the sample, which was subjected to mild alkaline trans-methylation with 1 mL of KOH/MeOH at 37 °C for 15 min, followed by cooling to room temperature. A 2 mL aliquot of 4:1 n-hexane/chloroform was added, and the sample was then neutralized with 1 mL of 1 M acetic acid. Subsequently, 2 mL of Milli Q water was added, and the phases were separated by centrifugation. The upper hexane layer was removed, and this step was repeated with fresh 2 mL aliquots of 4:1 n-hexane/chloroform. The combined hexane fractions were concentrated under compressed N_2_, and the fatty acids were re-solubilized with chloroform and analyzed.

## 4. Conclusions

In this study, we examined the effect of oxacillin combined with the fatty acid palmitic acid and the surfactant span85 on clinical strains, due to their antibacterial characteristics. To discover the optimal condition, we used the Box–Behnken design and response surface methodology analysis. We then proposed several conditions that were optimal to kill highly resistant clinical strains with very low concentrations of oxacillin. In addition, we showed that it is possible to kill more resistant strains, such as SCCmec type II strains, by increasing the oxacillin concentration.

To elucidate the reasons for the high resistance of clinical strains, PLFA analysis was conducted, and the results revealed different patterns of membrane fatty acid composition: more resistant strains contained a higher ratio of odd-chain fatty acids, such as 12-methyltetradecanoic acid (anteiso-C15:0) and 14-methylhexadecanoic acid (anteiso-C17:0). Although they may not be directly linked to the higher resistance of clinical samples and the effectiveness of a simple combination in killing all the strains, the different PLFA patterns appeared to be responsible for the higher resistance, based on the interaction between fatty acids and surfactants, which affected the membranes.

Our results showed that by combining oxacillin with palmitic acid and span85, the same level of antibacterial effects could be achieved with a lower concentration of oxacillin, thereby reducing the possibility of the strain acquiring drug resistance.

In conclusion, our data suggested a possible recycling strategy of safe antibiotics at present, in which their efficacy against resistant bacteria is increased via combined use with effective molecules.

## Figures and Tables

**Table 1:** Coded and real values of factors in the Box–Behnken experimental design.

Factor	Level of Factor		
	−1	0	1
Oxacillin (X_1_, μg/mL)	0	50	100
Palmitic acid (X_2_, mg/mL)	0	0.75	1.5
Span85 (X_3_, %)	0	0.25	0.5

**Table 2:** Box–Behnken experimental design matrix with experimental values of the cell growth of the Δ*agr* strain.

Runs	Oxacillin (μg/mL)	Palmitic Acid (mg/mL)	Span85 (% *v*/*v*)	OD (595 nm)
1	0	0	0.25	1.07 ± 0.03
2	100	0	0.25	0.12 ± 0.03
3	0	1.5	0.25	0.10 ± 0.03
4	100	1.5	0.25	0.00 ± 0.00
5	0	0.75	0	0.02 ± 0.02
6	100	0.75	0	0.02 ± 0.00
7	0	0.75	0.5	0.44 ± 0.00
8	100	0.75	0.5	0.00 ± 0.00
9	50	0	0	0.51 ± 0.07
10	50	1.5	0	0.10 ± 0.00
11	50	0	0.5	0.43 ± 0.02
12	50	1.5	0.5	0.05 ± 0.03
13	50	0.75	0.25	0.08 ± 0.02
14	50	0.75	0.25	0.01 ± 0.00
15	50	0.75	0.25	0.07 ± 0.00

**Table 3:** Analysis of variance for the selected model.

Source	DF	SS	MS	F-Value	Prob > F
Model	6	3.36120	0.56020	55.67	0.000
Error	38	0.38242	0.01006		
Total	44	3.74362			

DF, degree of freedom; SS, sum of squares; MS, mean squares.

**Table 4:** Optimized sets of variables using response optimizer.

Entry	Oxacillin (μg/mL)	Palmitic Acid (mg/mL)	Span85 (%)
#1	15	1.3	0.1
#2	50	1	0.08
#3	100	0.3	0.4

**Table 5:** Types of clinically isolated strains and oxacillin minimum inhibitory concentration (MIC) as well as their characteristics.

Name	Type	SCCmec Type	Oxacillin MIC (μg/mL)	Spa Type	MLST (ST)
LAC	MRSA	IV	20	t008	8
2065	MRSA	III	1024	t037	239
6230	MRSA	IV	128	t324	72
6288	MRSA	III	1024	t037	239
7557	MRSA	II	1024	t9353	5
7875	MRSA	IV	128	t664	72
8471	MRSA	II	1024	t9353	5
9291	MRSA	II	1024	t601	5
12779	MRSA	II	1024	t2460	5
14278	MRSA	II	1024	t9353	5
14459	MRSA	IV	1024	t324	72
